# Hyposkillia as a cause of delayed diagnosis of acute appendicitis in the second half of pregnancy

**DOI:** 10.1186/1753-6561-6-S4-P12

**Published:** 2012-07-09

**Authors:** A Rodionova

**Affiliations:** 1 I.M. Sechenov First Moscow State Medical University, Moscow, Russia

## Introduction

The incidence of acute appendicitis in pregnancy is 1:2000 (1:700 ÷ 3000); 60% suffer from acute appendicitis in the first half of pregnancy. When perforated appendicitis complicated by diffuse peritonitis, maternal mortality rate reaches 16.7%, with perinatal loss in the 19,4-50%. Explainable limitation application of instrumental diagnostic methods at different stages of pregnancy makes to rely more on clinical experience of doctor. However, the ubiquitous phenomenon observed by the lack of clinical skills (hyposkilliya) sometimes leads to tragic mistakes.

## Aims

1) improve practical skills of students' in the diagnosis of acute appendicitis in pregnant women; 2) identify the symptoms of acute appendicitis in pregnant women in the first and the second half; 3) the ultimate goal of this study is the reduction of maternal and perinatal mortality.

## Methods

Based on our experience and literature data, we identified 32 symptoms of acute appendicitis in pregnant women. Retrospectively analyzed medical histories of pregnant patients with acute appendicitis.In the first half (up to 20-21 weeks) pregnancy was observed 73% of patients in the second-27%. On the first day since the disease come to the clinic 51% in the second 27%, the third-22%.

## Results

Of 32 existent symptoms we identified 3 of the most important. For the first half of pregnancy were typical symptoms that characterize the local changes (muscular protection of the right iliac region, palpation and percussion sorenessabdominal wall infiltration in the pelvis, hypertonicity of the uterus), their reliability was 98%.The main diagnosis of acute appendicitis in the second half of pregnancy should take into account evidence of systemic response to inflammation (fever, tachycardia, high leukocytosis, shift to the left of leukocyte formula).Taken into account as nausea, repeated vomiting, the weakening of intestinal motility and diarrhea.Typical local symptoms of acute appendicitis are the symptoms of Brands, Michelson, Ridvan.

## Conclusions

1) The study showed the reliability of physical signs of acute appendicitis in the first half of pregnancy- 98%. The complementary physical examination in the later stages of gestation ultrasound diagnosis helped to establishment the correct diagnosis in 90%; 2) In our opinion, the implementation of the study will lead to a significant reduction of maternal and perinatal mortality.

